# Implementation of Machine Learning Applications in Health Care Organizations: Protocol for a Systematic Review of Empirical Studies

**DOI:** 10.2196/47971

**Published:** 2023-09-12

**Authors:** Vittoria Ardito, Giulia Cappellaro, Amelia Compagni, Francesco Petracca, Luigi Maria Preti

**Affiliations:** 1 Center for Research on Health and Social Care Management (CERGAS) SDA Bocconi School of Management Milan Italy; 2 Department of Social and Political Sciences Bocconi University Milan Italy

**Keywords:** artificial intelligence, barriers, facilitators, health care organization, implementation, machine learning

## Abstract

**Background:**

An increasing interest in machine learning (ML) has been observed among scholars and health care professionals. However, while ML-based applications have been shown to be effective and have the potential to change the delivery of patient care, their implementation in health care organizations is complex. There are several challenges that currently hamper the uptake of ML in daily practice, and there is currently limited knowledge on how these challenges have been addressed in empirical studies on implemented ML-based applications.

**Objective:**

The aim of this systematic literature review is twofold: (1) to map the ML-based applications implemented in health care organizations, with a focus on investigating the organizational dimensions that are relevant in the implementation process; and (2) to analyze the processes and strategies adopted to foster a successful uptake of ML.

**Methods:**

We developed this protocol following the PRISMA-P (Preferred Reporting Items for Systematic Review and Meta-Analysis Protocols) guidelines. The search was conducted on 3 databases (PubMed, Scopus, and Web of Science), considering a 10-year time frame (2013-2023). The search strategy was built around 4 blocks of keywords (artificial intelligence, implementation, health care, and study type). Based on the detailed inclusion criteria defined, only empirical studies documenting the implementation of ML-based applications used by health care professionals in clinical settings will be considered. The study protocol was registered in PROSPERO (International Prospective Register of Systematic Reviews).

**Results:**

The review is ongoing and is expected to be completed by September 2023. Data analysis is currently underway, and the first results are expected to be submitted for publication in November 2023. The study was funded by the European Union within the Multilayered Urban Sustainability Action (MUSA) project.

**Conclusions:**

ML-based applications involving clinical decision support and automation of clinical tasks present unique traits that add several layers of complexity compared with earlier health technologies. Our review aims at contributing to the existing literature by investigating the implementation of ML from an organizational perspective and by systematizing a conspicuous amount of information on factors influencing implementation.

**International Registered Report Identifier (IRRID):**

DERR1-10.2196/47971

## Introduction

### Rationale

After a period of so-called artificial intelligence (AI) winter, AI has re-emerged into the scientific and public consciousness by virtue of new technological breakthroughs and marketed solutions [1]. Recent machine learning (ML) applications have expanded the frontiers of AI. In this scenario, finding an operational definition of AI and ML is challenging. While the term AI has been embraced for regulatory purposes [2], various technical domains view it as a descriptor for the latest advancements in computer science. Consequently, as AI becomes more pervasive and integrated into mainstream applications, experts gradually disassociate it from its original designation [3]. ML instead refers to the subset of AI encompassing all the non–knowledge-based techniques that automatically learn from data without being explicitly programmed [4,5]. Recent advances in ML promise remarkable improvements in medical diagnosis and prognosis [6,7], generating tremendous enthusiasm surrounding these innovations. The use of ML in clinical environments has hitherto experienced diverse adoption patterns, with the field of radiology raising considerable attention due to the notable capability of ML (in particular, deep learning [DL]) models to extract valuable information from medical images [7]. Other clinical areas are also a fertile environment for ML-based applications, as their outcome prediction capabilities are expected to help health care systems improve the quality of care and use resources more accurately and efficiently [8].

Regulatory science has tried to keep up with these advances by adopting a permissionless innovation approach with low barriers for the entry of new algorithms [9]. Despite the European Union and the United States regulating AI with distinctiveness [10], regulatory approaches to ML are still under definition, ultimately impacting on the access strategies of developers [11] and contributing to delaying the deployment of ML in clinical practice and in specific settings.

Previous literature has well documented the implementation of expert systems and knowledge- or logic-based applications [12], all included in the definition of AI proposed by the High-Level Expert Group on Artificial Intelligence appointed by the European Commission [13]. Instead, ML-based applications assumed a prominent role only recently, thanks to greater data availability and computing capabilities [14]. Nevertheless, due to the incremental complexity of ML-based applications [12], their incorporation into health care processes is fraught with different challenges that need to be organically steered by various stakeholders [15]. ML-based applications that encompass the characteristics of autonomy, inscrutability, and learning [16] hence need further investigation from the implementation science community.

Recent scholarly contributions have shown that a theoretical understanding of the dimensions and dynamics associated with the implementation of AI in health care organizations is still in its infancy [14]. Empirical knowledge about the implementation of AI comes mostly from systems with a low level of autonomy and learning (ie, knowledge-based applications) and looks at either clinical effectiveness, technical efficiency, or regulatory and legal issues of such technologies, overlooking the organizational aspects [17]. For this reason, our work focuses exclusively on non–knowledge-based ML, namely tools that use some form of either supervised or unsupervised learning, and not on traditional knowledge-based AI algorithms.

Research on ML implementation has been predominantly conceptual in nature, with the identification of distinguishing issues in the development and dissemination in clinical practice, such as the opacity or black-box effect, and concerns surrounding equity, data privacy, and security [7,18]. Conversely, the consequences that such characteristics have on the implementation of ML in health care organizations have been poorly investigated empirically. A recent scoping review by Chomutare and colleagues [19] adopted a mixed deductive-inductive approach to extract barriers and facilitators to the implementation of ML and map them onto the Consolidated Framework for Implementation Research (CFIR) [20], identifying generalizability, interoperability, and data quality as the main barriers and engagement in the implementation process as the main facilitator [19]. However, additional inquiry is needed to gauge whether the current body of empirical studies of ML adequately acknowledges distinguishing implementation challenges such as trust and explainability and to analyze which implementation strategies have been adopted to address them.

### Objectives

Given the potential disruptiveness of recent developments of ML in health care and the lack of knowledge about how to overcome the organizational challenges associated with their implementation, our research objectives are to identify and explore cases of real-life implementation of ML within health care organizations as documented in the literature and to synthesize the processes adopted to foster a successful implementation of ML. We plan to conduct a systematic literature review with the following goals in mind:

1. To examine the stages and characteristics of implementation processes described in empirical studies on ML under the lens of implementation science, namely the scientific study of methods to promote the uptake of evidence-based innovations into routine practice [21]. Specifically, the CFIR will be adopted as a practical theory-based guide for assessing the implementation outcome and complemented with implementation considerations associated with the distinctive features of ML.
2. To analyze the observed or suggested determinants of successful uptake of ML, thereby identifying the implementation processes and strategies currently adopted by health care organizations.

## Methods

### Study Design

This systematic review protocol follows the PRISMA-P (Preferred Reporting Items for Systematic Review and Meta-Analysis Protocols) statement [22,23] and was registered within the PROSPERO (International Prospective Register of Systematic Reviews) on March 3, 2023 (registration number 403873).

### Eligibility Criteria

This review will be focused on studies that investigate aspects related to the implementation of ML-based applications in health care organizations. For the purposes of this review, by implementation, we define the “active and planned effort to mainstream innovation within an organization” [24], while the term “health care organization” refers to entities that deliver health services, including hospitals (general, community, teaching, or research hospitals), outpatient centers, and primary care and public health facilities. Studies will be selected based on the eligibility criteria described below and summarized in Textbox 1.

**Eligibility criteria.**

**Inclusion criteria**

- Study design: empirical studies based on implementation research designs (experimental or quasi-experimental, observational, hybrid, and simulation study designs)
- Intervention: implementation of machine learning (ML)–based applications including considerations on the inner setting and/or implementation process domains
- Stakeholder groups: ML-based applications used at least by health care professionals
- Settings: hospitals, outpatients, and other community care settings

**Exclusion criteria**

- Study design: effectiveness research study designs, literature reviews, commentaries, editorials, opinion articles, study protocols, studies collecting perceptions on implementation, and unrelated to specific ML-based applications
- Intervention: implementation of knowledge-based applications
- Stakeholder groups: ML-based applications targeting patients and other nonclinical stakeholders (eg, caregivers, policy makers, and regulators) only

### Study Design and Publication Type

We will select peer-reviewed empirical studies documenting the implementation of ML-based applications, thereby including experimental or quasi-experimental, observational, hybrid, and simulation study designs [25]. In the context of implementation research, experimental studies typically aim to evaluate the impact of specific strategies to improve implementation, whereas observational designs examine the implementation in service delivery using approaches that are quantitative, qualitative (eg, semistructured interviews with involved end users), or mixed methodologies. We will also consider hybrid designs, which blend the components aimed at testing clinical effectiveness with those that analyze the implementation process by investigating potential barriers and facilitators to the implementation and assessing outcomes such as adoption, reach, acceptability, and fidelity [26]. We will exclude comparative effectiveness study designs (such as randomized controlled trials that only aim at generating clinical evidence and do not imply organization-wide implementation), as well as nonempirical designs, including editorials, commentaries, opinion articles, and other published reviews of the literature. Works based on surveys and interviews with subject matter experts will be excluded if they broadly investigate issues concerning the adoption of ML-based applications and do not report on the implementation process of specific technologies in a given organizational setting.

### Intervention

Studies will be considered eligible if they analyze the implementation of tools based on ML in health care organizations and discuss the associated processes, context, barriers, or facilitators. As part of ML, we will include both DL techniques, unanimously considered as a subset of ML [5], and natural language processing models when based on ML. The latter, by their very nature, do not necessarily involve learning techniques. However, DL techniques have been widely used to overcome the limitations of symbolic approaches applied to natural language processing, and most of its recent development is unanimously attributed to DL approaches [27-29].

Based on the constructs of the updated CFIR [20], only articles including considerations on either the inner setting in which the innovation has been adopted or the activities and strategies being used to implement the innovation will be included. Studies only focusing on the clinical implications of the implementation or based on logic- and knowledge-based applications (expert systems) will be excluded.

### Stakeholder Groups

We will include studies focused on ML-based applications available for use by health care professionals. Applications used by patients, caregivers, health care administrators, and policy makers will only be included if they are also used by clinicians and other health care professional groups.

### Setting

We will include studies with a spotlight on the implementation of ML-based applications in clinical practice, biomedical research, and public and global health, as per the classification provided by the European Parliament [30]. All possible organizational settings related to these practices will be considered, including inpatient units, emergency departments, outpatient settings, and primary care. ML-based applications used in administrative settings will be excluded.

### Time Frame

Considering the rapid evolution and changes in ML-based applications, we will only consider studies published in the past 10 years (since 2013).

### Information Sources

Literature searches were conducted in MEDLINE (PubMed), Scopus, and Web of Science and replicated in top-tier management journals (ie, Academy of Management Journal, Organization Science, MIS Quarterly, Journal of Management Studies, Organization Studies, and Academy of Management Annals). In addition, the reference list of all included studies and of the reviews identified will be scanned to ensure that any relevant work is captured. Gray literature was not considered.

### Search Strategy

The search strategy was developed, including a combination of keywords around four main concepts: (1) AI, (2) implementation, (3) health care, and (4) study type.

The list of search concepts and associated terms used is provided in Textbox 2.

The exact search stream was developed by the research team following an iterative process. The general term “artificial intelligence” has been used to encompass studies that address the terms AI and ML as synonymous. The search strategy was initially finalized for PubMed and then adapted to the syntax and subject headings of the other databases. The initial search was performed in April 2023.

**Concepts to be used to define the search string.**

**Main search concepts, to be combined using “AND”**

- Artificial intelligence
- Implementation
- Healthcare
- Study type

**Search concepts synonyms, to be combined using “OR”**

- Artificial Intelligence, Machine Learning, Deep Learning, Natural Language Processing
- Implementation, Adoption, Diffusion, Uptake
- Healthcare, Health*, Therap*, Patient*, Clinic*, Care, Professional, Medic*, Doctor, Hospital, Life Science, Pharma
- Empirical, quantitative, trial, qualitative, interview*, case stud*, case report, field stud*, field research, mixed-method*, mixed method*, focus group*, observational stud*, hybrid design, hybrid stud*, trial

### Study Records

#### Data Management

The records retrieved from the literature search were uploaded and merged into RefWorks, a reference management tool that simplifies the process of research and collaboration among the review team. Duplicates were removed, and the identified list of records was extracted into an Excel (Microsoft Corp) spreadsheet for title and abstract screening.

#### Selection Process

A total of 2 researchers (VA and LMP) screened the first 100 retrieved articles based on titles and abstracts. Interrater agreement was measured using kappa statistics [31]. Once aligned on the eligibility criteria, the remaining papers were screened independently by the review team (VA and LMP) based on titles and abstracts. Disagreement over final inclusions was solved with a third researcher (FP). Studies deemed eligible for full-text reading will be assessed in depth (VA, LMP, and FP). Disagreements will be solved by dialogue with 2 other researchers (GC and AC). The entire research team will read all the studies eventually included in the analysis.

#### Data Collection Process

The data collection process will be performed by 3 reviewers (VA, LMP, and FP). Data will be extracted using an ad hoc Excel (Microsoft Corp) spreadsheet, preliminarily developed by the research team. To ensure consistency across reviewers, the extraction sheet will be tested by each reviewer and possibly recalibrated before starting the data collection process. Disagreements will be resolved by discussion, and 1 of 2 arbitrators (GC and AC) will adjudicate unresolved disagreements. Information on the implementation process of ML-based applications will be collected, as specified in the following section.

#### Data Items

Data items to be extracted from the selected studies will include the following fields, based on preexisting, established classifications or schemes, where possible:

1. Demographic information: study identifiers, authors, journal outlet, publication year, and keywords.
2. Methodology: study design.
3. Setting: country, organizational setting (eg, hospital inpatient, emergency department, and primary care), organizational nature (eg, public and private), and unit of implementation of the ML-based application.
4. Implementation stages: stages of the implementation process described in the study, adapted based on Aarons et al [32].
5. Intervention: name and description of the ML-based application, level of integration with other technologies, main practice (ie, clinical practice, biomedical research, public and global health, and health care administration) [30], steps of the clinical workflow impacted by the ML-based application [33], ML capabilities (eg, recognizing sounds vs images vs texts) [34], type of learning system (ie, supervised vs unsupervised vs semisupervised learning) [35,36], level of autonomy that the ML-based application foresees (from data presentation only to full autonomation following the classification by Bitterman and colleagues [37]), underlying data fed in the ML-based application (ie, quantity, quality, and data inputs), and previous evaluation of effectiveness [14].
6. Individuals: actors involved in the development and implementation of the ML-based application.
7. Inner setting: factors related to the organizational context in which the innovation is implemented, including both persistent general characteristics of the organization and properties specific to the delivery of the innovation. These include implementation capacity and readiness, as well as ML-specific domains such as the consideration of ethical concerns [38] and computational resources (ie, any references to the availability of substantial computing power, either proprietary or through cloud-based analytics, and organizational policies or strategies that have favored data storage and processing) [39].
8. Outer setting: considerations on the setting in which the inner setting exists, such as legislative, regulatory, accreditation, and reporting authorities and aspects [40], partnerships and connections with external entities, and external pressure from either social movements, peer organizations already adopting the technology, or benchmarking metrics [20].
9. Implementation process, including activities and strategies used to implement, assimilate, and routinize the innovation, encourage participation, and collectively discuss the success of the process.
10. Barriers and facilitators explicitly cited in the study.
11. Outcomes: observed, measurable outcome domains and metrics associated with the implementation process.

#### Data Synthesis

A narrative analysis will be adopted to synthesize the findings from the included studies, as the characteristics of the implementation processes will be tabulated and summarized through descriptive statistics according to the implementation-science domains selected for data collection. Given the objectives of this systematic review, the wide variety in the study designs and outcome domains considered, and the prospected predominance of qualitative studies, no quantitative synthesis of the results will be performed.

## Results

The systematic review is ongoing. As of June 1, 2023, we have finalized the identification of records and the title and abstract screening of the 3520 records that met our search criteria. A full-text review is currently underway and will be completed by September 2023. After data synthesis, we plan to disseminate the study results by submitting the corresponding manuscript to a peer-reviewed journal in November 2023.

## Discussion

ML-based applications have proliferated exponentially in recent years and demonstrated to have high potential both to streamline the clinical workflows and to improve patient outcomes. Yet, available studies have focused mainly on the technical components of ML-based applications and on their clinical efficacy, while the organizational preconditions or determinants needed to facilitate or sustain their implementation have been overlooked, emphasizing the need to identify effective strategies and foster the use of theories, models, and frameworks to facilitate implementation [41].

Therefore, our research will systematically review and synthesize the evidence of empirical studies reporting on implemented ML-based applications that are used in the real world. To comprehensively investigate implementation factors at the organizational level, ML-based applications that impact the activities of health care professionals at large will be considered.

In this context, the domains of the updated CFIR will be used as a practical tool to guide the data extraction and interpretation of the organizational factors that drive implementation, adopting the theoretical lens of implementation science. As such, we expect that our review will contribute to the field of implementation science research, a field that seeks to systematically close the gap between what we know and what we do (ie, the know-do gap) [21], adding insightful knowledge to the implementation science literature on ML.

The strengths of our prospective literature review are that it will follow a standardized methodological approach by adhering to systematic review guidelines (ie, PRISMA statements and checklist for systematic reviews) [42] and that it will aim at systematizing a conspicuous amount of information on factors influencing implementation. On the contrary, potential limitations lie both in language barriers, as only articles published in English will be scrutinized, and on the exclusion of the gray literature.

This work is part of larger research whose overarching aim is to develop a conceptual framework for the implementation of ML in health care organizations in an effort to support the adoption and deployment of health care innovations. In perspective, the theoretical basis of this work will therefore pave the way for the later conceptualization of an implementation framework that will stem from the available implementation science frameworks, but will be tailored to also account for the distinctive features of ML. This process will be additionally informed by the engagement of expert stakeholders employed in health care organizations, who will provide qualitative inputs based on their real-life experience with ML to fine-tune and finalize the framework. All in all, starting with the current review, the aim of our research is twofold: building a practical tool for health care practitioners while transitioning toward the use of ML-based applications within their organization, while also contributing theoretically to management and innovation theories.

## Abbreviations

AI: artificial intelligence
CFIR: Consolidated Framework for Implementation Research
DL: deep learning
ML: machine learning
MUSA: Multilayered Urban Sustainability Action
PRISMA-P: Preferred Reporting Items for Systematic Review and Meta-Analysis Protocols
PROSPERO: International Prospective Register of Systematic Reviews

## Data Availability

Data sharing is not applicable to this article as no data sets were generated or analyzed for this research protocol. All data extracted from the literature and generated during the prospective systematic review will be included in the published article or made available upon request.
